# Shortening “the Road” to Improve Engagement with HIV Testing Resources: A Qualitative Study Among Stakeholders in Rural Uganda

**DOI:** 10.1089/apc.2020.0235

**Published:** 2021-02-09

**Authors:** Kathryn Broderick, Matthew Ponticiello, Doreen Nabukalu, Patricia Tushemereirwe, Gabriel Nuwagaba, Rachel King, Juliet Mwanga-Amumpaire, Radhika Sundararajan

**Affiliations:** ^1^ Weill Cornell Medical College, New York, New York, USA.; ^2^ Department of Global and Public Health Sciences, Cornell University, Ithaca, New York, USA.; ^3^ Department of Pediatrics and Child Health, Mbarara University of Science and Technology, Mbarara, Uganda.; ^4^ Global Health Sciences, University of California, San Francisco, San Francisco, California, USA.; ^5^ Center for Global Health, Weill Cornell Medicine, New York, New York, USA.; ^6^ Department of Emergency Medicine, Weill Cornell Medicine, New York, New York, USA.

**Keywords:** qualitative research, patient acceptance of health care, health service acceptability, HIV/AIDS, Uganda, traditional medicine

## Abstract

In HIV-endemic areas, traditional healers are frequently used with, or instead of, biomedical resources for health care needs. Studies show healers are interested in and capable of supporting patients in the HIV care cascade. However, adults who receive care from healers have low engagement with HIV services. To achieve epidemic control, we must understand gaps between the needs of HIV-endemic communities and the potential for healers to improve HIV service uptake. This study's objective was to characterize stakeholder perspectives on barriers to HIV testing and approaches to mitigate barriers in a medically pluralistic, HIV-endemic region. This study was conducted in Mbarara District, a rural area of southwestern Uganda with high HIV prevalence. Participants included HIV clinical staff, traditional healers, and adults receiving care from healers. Fifty-six participants [*N* = 30 females (52%), median age 40 years (interquartile range, 32–51.5)] were recruited across three stakeholder groups for minimally structured interviews. Themes were identified using an inductive, grounded theory approach and linked together to create a framework explaining stakeholder perspectives on HIV testing. Stakeholders described the “road” to HIV testing as time-consuming, expensive, and stigmatizing. All agreed healers could mitigate barriers by delivering HIV testing at their practices. Collaborations between biomedical and traditional providers were considered essential to a successful healer-delivered HIV testing program. This work describes a novel approach to “shorten the road” to HIV testing, suggesting that traditional healer-delivered HIV testing holds promise to expand uptake of testing among communities with limited access to existing programs.

## Introduction

Uganda is an HIV-endemic region and is yet to reach the United Nations' 95-95-95 benchmarks for an AIDS-free generation.^1^ In some rural areas, for example, only one-third of sexually active adults have tested for HIV in the prior 12 months.^2^ While increased capacity at biomedical facilities has improved uptake, most notably through antenatal care,^3,4^ low engagement with HIV testing remains a major barrier to epidemic control.^5–7^

Uganda is medically pluralistic, where people frequently use traditional healers with, or in place of, biomedical care.^8,9^ Throughout sub-Saharan Africa, healers have demonstrated interest in supporting patients through the HIV continuum of care.^10–13^ However, adults receiving care from traditional healers have suboptimal HIV service utilization. Using a healer has been associated with prolonged time to HIV diagnosis^14^ in addition to reduced antiretroviral therapy (ART) adherence among people living with HIV.^15^ Further research is needed to harmonize the needs of HIV-endemic communities with traditional healers' capacity to support HIV services engagement.

This study addresses this gap in knowledge by exploring community perspectives on improving uptake of HIV testing in rural southwestern Uganda. We conducted qualitative interviews with three groups of stakeholders to explore concepts pertinent to improving HIV testing programs for communities where traditional healers are commonly utilized.

## Methods

### Study setting

Mbarara District in southwestern Uganda is a rural region located 270 km from Kampala with ∼475,000 residents. HIV prevalence is 7.9%, higher than the national prevalence of 5.7%.^2,16^ Mbarara Regional Referral Hospital (MRRH) is a teaching hospital for Mbarara University of Science and Technology. MRRH runs the Immune Suppression Syndrome (ISS) clinic, the District's largest HIV clinic, which provides free HIV care. The ISS clinic and other HIV testing facilities are located in the Mbarara Township.

Approximately 80% of Ugandans utilize traditional healers.^8^ Ugandan traditional healers practice four distinct specialties: herbal medicine (herbalists), spiritual healing (spiritualists), prenatal care, labor, and delivery (birth attendants), and treatment of broken bones (bonesetters). Healers who work close to markets and other businesses often have practices that resemble medical clinics. In rural settings, some healers provide care for patients in structures frequently located adjacent to the healer's residence.

### Sampling and recruitment

Purposive sampling was used to identify participants representing three stakeholder groups in Mbarara District: (1) HIV clinical staff, (2) traditional healers; and (3) adults receiving care from traditional healers. Eligible participants were aged ≥18 years. HIV clinic staff were employed by the ISS clinic. Two authors (D.N. and P.T.) presented a summary of the study at an ISS staff meeting. Names of employees representing a range of clinic positions (physician, nurse, counselor, laboratory technician) were provided by the clinic director. Authors D.N., P.T., and G.N. contacted these employees to invite them to participate.

In early 2018, we conducted a census to identify all traditional healers practicing in Mbarara District, where locations, specialty, and gender were recorded. For this study, we recruited healers from this list with practice locations within a 30-kilometer radius of Mbarara Township. Healers and their clients were recruited in person, and sampled with the intent to achieve gender balance and proportional representation from each of the healer specialties.

Sample size for clinical staff was set at 12 clinical staff participants, and 20 individuals per group for healers and clients. Prior research indicates that a sample size of 12 participants is sufficient to reach data saturation within homogenous groups.^17^ Healers and their clients were anticipated to be heterogeneous, and we estimated data saturation at 20 participants per group. Data saturation was reached after 20 interviews in these 2 groups, but 22 interviews per group were conducted due to transcription lag time. Overall, 56 minimally structured interviews were completed.

### Data collection

Between August 2018 and April 2019, healers, clients, and ISS staff members were invited to participate in a single interview. Three Ugandan coauthors (D.N., P.T., G.N.) conducted the interviews. D.N. and P.T. are female, and G.N. is male. All are fluent in Runyankole (the local language) and English. Interviewers did not have prior relationships with participants. Clients of healers were recruited following treatment, to avoid giving the impression that study participation was required to receive treatment by the healer. HIV clinic staff interviews were conducted at their workplaces. All participants invited for interviews agreed to participate.

Interviews were conducted in Runyankole, in private locations, lasting ∼60 min, and audio-recorded. Interview guides were used to ensure consistency of topics across interviews and exploration of novel concepts. Interview topics included engagement with HIV testing, biomedical and traditional medicine utilization, and strategies to improve HIV testing uptake. Guides were pilot tested with one member of each stakeholder group before initiation of the study, and not included in the study data set. Transcripts were not returned to participants for comment, but preliminary results were presented to ISS clinical staff in June 2019.

### Data analysis

Deidentified transcripts were transcribed and translated into English by the interviewer. English transcripts were reviewed by author R.S. within 72 h of transcription for quality. Author J.M.-A. (fluent in English and Runyankole) spot-checked transcripts to ensure translational integrity. R.S. and K.B. independently reviewed transcripts to create a list of codes relevant to barriers to HIV testing, and avenues for improvement. Codes were generated in an open-coding manner and refined using the constant comparison method.^18^ Through discussion and consensus, these two authors developed a final set of codes. Final codes were grouped into themes and analyzed using a grounded theory approach^18–20^ to produce a framework explaining stakeholder perspectives on HIV testing and solutions to improve uptake.

### Ethics approvals

This study was approved by the Weill Cornell Medicine Institutional Review Board (Protocol 18-03019105), Mbarara University of Science and Technology Institutional Review Board (Protocol 16-/01-17), and Ugandan National Council on Science and Technology (Protocol SS-4338). All participants provided written informed consent. Traditional healers provided consent for recruitment of patients at their practice locations. Participants received household staples valued at 10,000 Ugandan Shillings (UGX, ∼2 USD) as remuneration on interview completion. The study followed the consolidated criteria for reporting qualitative research (COREQ) reporting guidelines.^21^

## Results

### Characteristics of study participants

Fifty-six interviews were conducted among traditional healers (*N* = 22), clients of healers (*N* = 22), and HIV clinic staff (*N* = 12). Summary characteristics are shown in Tables 1 and 2. The majority were female (*N* = 30, 54%). Healers tended to be older than HIV staff and clients. Most healers and clients had primary school education or less, and most HIV staff had at least a bachelor's degree.

**Table 1.** Characteristics of Participating Healers and Their Clients

Characteristics	Healers (*n* = 22)	Clients of healers (*n* = 22)
Female, no. (%)	13 (59.1)	12 (54.5)
Age (years), median (IQR)	52 (38.5–66)	34.5 (28.5–46)
Highest level of education, no. (%)	Primary school or less, 15	Primary school or less, 19
	Secondary school, 6	Secondary school, 2
	Diploma or higher, 1	Diploma or higher, 1
Healer specialty, no. (%)	Spiritualist, 6 (27)	Spiritualist, 5 (23)
	Herbalist, 6 (27)	Herbalist, 6 (27)
	Traditional birth attendant, 5 (23)	Traditional birth attendant, 5(23)
	Bonesetter, 5 (23)	Bonesetter, 6 (27)
Received an HIV test in the last 12 months, no. (%)	12 (54.5)	14 (63.6)

IQR, interquartile range.

**Table 2.** Characteristics of Participating HIV Clinic Staff

Characteristics	Clinical staff (*n* = 12)
Female, no. (%)	5 (41.7)
Age (years), median (IQR)	35.5 (31–43.75)
Highest level of education, no. (%)	Bachelor's degree, 6 (50)
	Master's degree, 2 (17)
	Professional degree, 4 (33)
Clinical position, no. (%)	Laboratory technologist, 3 (25)
	Counselor, 2 (17)
	Nurse, 3 (25)
	Physician, 4 (33)

IQR, interquartile range.

### Overview

We describe stakeholder perspectives on improving engagement with HIV testing resources. First, we present factors contributing to low uptake of existing HIV services, illustrating metaphorical and physical roadblocks to HIV testing. Second, we examine an avenue to improve HIV testing uptake, specifically by involving traditional healers in the delivery of HIV testing. Finally, we describe elements essential to a healer-delivered HIV testing program.

### Roadblocks to HIV testing services

**FIG. 1.**
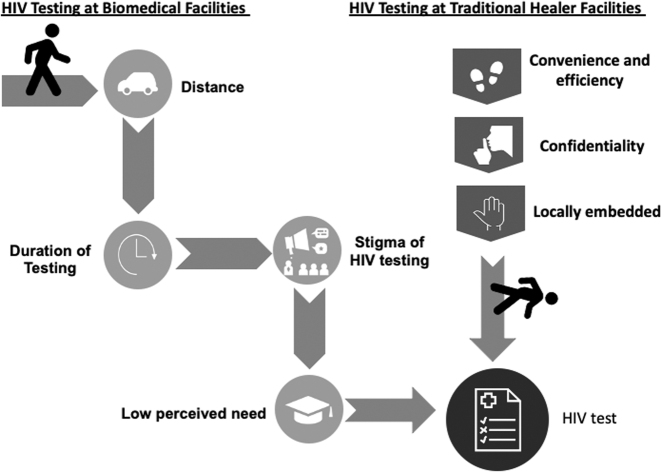
On the left, the “road” to HIV testing is conceptualized with impeding factors. On the right, the shortened “road” is conceptualized through a healer-delivered service, with factors facilitating engagement with HIV testing services.

Four themes explained the low uptake of existing HIV testing services: (1) distance; (2) duration of testing; (3) HIV stigma; and (4) low perceived need for testing (Fig. 1).

#### Distance from HIV testing services

The distance from HIV testing locations and the state of the roads to facilities were described as significant barriers. Poor transportation infrastructure presented a challenge for participants to access testing services. Travel to testing facilities required motorized transportation or hours of walking.

The roads to the nearest health facilities are very poor. The roads are in a sorry state.—Traditional healer, Male 62 years old

#### Duration of HIV testing

HIV testing was also described as expensive and time-consuming with clinics being understaffed and underresourced. Facilities ran out of testing supplies in some instances and turned people away. Participants also reported difficulty paying for transportation to clinics given competing household priorities.

Instead of wasting 2000UGX on transport [to the HIV testing facility], I better buy a kilo of maize flour for my family.—Client of traditional healer, Male 42 years old

#### HIV-related stigma

HIV-related stigma was a prominent barrier to engaging with existing HIV testing. Participants feared being seen at testing facilities because others may assume their status as positive.

Most people have stigma. They fear to come where there is HIV testing and counseling. They fear people to see them coming from HIV testing room… stigma is the biggest challenge people have.—HIV clinic worker, Female 28 years old

“Fear” of being recognized was also described when utilizing mobile HIV testing services in the community. Participants reported that seeking out an HIV test in any context associated an individual with stigmatized, high-risk sexual behaviors.

#### Low perceived need for testing

Finally, a low perceived need for HIV testing impeded engagement with resources. Participants lacked motivation to test due to the absence of symptoms, perceiving their risk of HIV and need for testing as low. The time and effort required to test were not justifiable if they were asymptomatic.

I find it unnecessary. Besides, I am too busy with my daily work. I have no time to spend on such things, and to be honest I do not find it useful … When not sick, why would one test for HIV?—Client of traditional healer, Male 42 years old

### Strategies to improve HIV testing

When describing an ideal HIV testing program, participants reported strategies to overcome aforementioned barriers. Across stakeholder groups, traditional healers were suggested to mitigate roadblocks, improving access to and uptake of HIV testing in three ways: (1) convenience and efficiency, (2) increased confidentiality, and (3) culturally concordant care (Fig. 1).

#### Convenience and efficiency

Participants predicted increased convenience and efficiency with HIV testing delivered at a healer's location.

It will also be good for the patient [to test here] since they don't have to go through the hassle of going to the health facility for testing, because that means more expenses, transport, time wasting, et cetera.—Traditional healer, Male 66 years old

Clients reported interest in receiving HIV testing in addition to treatment for whatever ailment had originally driven them to seek traditional care. Healers suggested that knowing a patient's HIV status could improve delivery of traditional care.

[HIV testing] can be very good if it's the first thing I do when a patient comes to my clinic, so that we rule out HIV immediately and start investigating other problems.—Traditional healer, Male 52 years old

#### Confidentiality

Second, participants believed that healers could mitigate HIV-related stigma through improved confidentiality. At a healer's location, no one would know that a client's visit was related to HIV services. The “fear to be recognized” at an HIV testing site was not anticipated to carry over to traditional healers.

A patient would visit a traditional healer to take an HIV test, while another patient would visit the healer for another condition, and you will not know what kind of different [service] each patient is seeking.—Client of traditional healer, Male 21 years old

#### Culturally concordant care

Finally, participants described traditional healers as locally embedded and culturally concordant. Many referred to traditional healers as trusted providers who could provide personalized care more effectively than biomedical clinical staff.

Some people may say that, for them, they don't believe in western medicine. They would rather go to a traditional healer than waste time coming here to the health facility.—HIV clinic worker, Male 28 years old

I know my clients very well and they confide in me. So, it may be easy for a client to open up to me, rather than the health worker.—Traditional healer, Female 70 years old

### Implementation of a traditional healer-delivered HIV testing program

Cooperation between stakeholders was considered essential for effective implementation of a traditional healer-delivered testing program, with two central aspects: (1) training and certification; and (2) collaboration between biomedical and traditional providers. HIV-related stigma was described as potential remaining barrier to testing.

#### Training and certification

While all stakeholder groups agreed that offering HIV testing at healers' locations would likely increase testing access and uptake, participants emphasized that services should align with biomedical expertise. A training and credentialing process was described as a means to ensure quality control and biomedical oversight of the HIV testing process.

When patients get to know that I was trained to test people for HIV and see a certificate allowing me to do so, they will not hesitate [to test here]—Traditional healer, Female 55 years old

However, lack of a shared knowledge base was suggested as a barrier to a successful program. Some HIV staff and healer clients were concerned that healers may reject biomedical tools, and would be unable to deliver HIV tests.

I doubt that a traditional healer can offer any support in HIV care … The traditional healer may not accept biomedical medicine, just like you don't accept traditional medicine.—Client of traditional healer, Male 48 years old

In contrast, healers uniformly reported that HIV should be “managed by biomedicine,” and acknowledged that they often felt misunderstood by biomedical providers.

#### Collaborations between traditional and biomedicine

Despite concerns over their differences, healers and HIV clinical staff expressed willingness to collaborate, suggesting that cooperative training sessions could be an effective means to overcome distrust and misunderstandings. *S*takeholders also acknowledged that trust between clients and healers could be leveraged to increase the reach of HIV testing services.

I think if he [the healer] is provided with all that he needs for testing, and knowledge on how to do counselling, they [healers] can really help us a lot.—HIV clinic worker, Female 31 years old

#### HIV-related stigma as a remaining barrier

Finally, participants noted that HIV-related stigma may remain a significant barrier to testing uptake. Healers believed that HIV was so stigmatized that patients may withhold their status from them.

I do not know why [patients] hide their HIV status from me, knowing that I cannot heal that sickness. Maybe they think that when they disclose, I will not attend to them?—Traditional healer, Male 33 years

Healers hoped that increased availability of a testing could normalize discussion of HIV status with clients. However, patients and HIV clinical staff were concerned that offering testing at a healer's location may be insufficient to overcome stigma, as individuals may want to hide their HIV status from their local community.

Local people may not be comfortable being tested by their own people.—HIV clinic worker, Male 48 years old

I even don't want him [the healer] to know that I am HIV infected.—Client of traditional healer, Female 30 years old

## Discussion

This study explored stakeholder perspectives on uptake of HIV testing in rural Uganda, where traditional healers play an important role in community health. We solicited perspectives from HIV clinic staff, traditional healers, and adults receiving care from traditional healers. Our findings illustrate existing barriers to HIV testing and suggestions for a healer-delivered HIV testing program. Our data show that “the road” to testing is arduous. Traveling to testing sites is expensive, and the process is time-consuming. Testing carries stigma, and the perceived need for testing is low if an individual feels healthy. We illustrate how traditional healers could expand HIV testing in rural communities. By including HIV testing at healer facilities, testing could be brought into routinely visited, convenient, and private spaces, which may carry less HIV-related stigma. Thus, healers could shorten the road to HIV testing.

Other community-based initiatives to deliver HIV testing have included HIV self-testing,^22,23^ home-based testing,^24,25^ and mobile outreach.^26,27^ While these initiatives have had some success expanding access to HIV testing in sub-Saharan Africa, scale-up has been limited due to difficulty reaching men and young adults,^28^ low literacy, and desire for professional counseling support.^29,30^ A healer-delivered intervention may overcome these barriers. A program delivered by healers would leverage the strength of the healer–client relationship and healers' social capital^9,31–33^ to deliver health counseling and HIV testing that would not require significant program infrastructure. Moreover, the potential for a healer-delivered service is well established in the literature,^11,34–36^ as is healers' willingness to participate in HIV program implementation.^12,37^

Our findings highlight key considerations for decentralizing HIV testing in rural communities. A healer-based testing service must be credible to clients and biomedical staff, especially because HIV testing is outside the scope of usual traditional care. Our data indicate that training and credentialing are essential for a program to be perceived as valid by those delivering tests and by those receiving the results. Although oral swab technology is new to much of sub-Saharan Africa, we suggest use of these point-of-care tests in a healer-delivered testing program, similar to “supervised” HIV self-testing initiatives.^38^ Oral swab tests are highly sensitive and specific, noninvasive, approved for layperson use,^39,40^ and endorsed by the Ugandan Ministry of Health.^41^ Importantly, traditional healers expressed belief in biomedical management of HIV, although some HIV staff and clients reported skepticism about harmonizing traditional and biomedical knowledge bases. Our results underscore the importance of relationship-building between biomedical and traditional providers to reduce distrust between these stakeholders.^42^

While our findings provide a strong framework for supporting a healer-delivered intervention, we—and many others—recognize that stigma remains a significant barrier to any effective HIV testing program.^43,44^ Many have noted the prevalence of stigmatizing attitudes among health care workers toward people living with HIV, and called for targeted stigma-reduction interventions in health care settings.^45,46^ We believe that such efforts should be inclusive of informal providers such as traditional healers, whose stigmatizing attitudes may also negatively impact health care engagement among clients.

This study has some limitations. Our sample did not include stakeholders from the Ministry of Health or District government. Nonetheless, qualitative data are meant to be hypothesis-generating and highly contextual. The nature of our data supports the validity of our findings within the study population. More research is needed to determine if our findings are generalizable beyond rural Uganda, and pilot studies of this approach are needed before moving to implementation at scale.

We have illustrated how the “road” to HIV testing is long, and propose a means to shorten the road via healer-delivered HIV testing. We hope these findings will inform the development of an implementation strategy that fosters collaboration between biomedical and traditional providers to successfully expand the uptake of HIV testing in endemic regions.
